# Cryptic diversity in *Astroblepus* (Siluriformes: Astroblepidae): Integrative taxonomy reveals evolutionary complexity in the Esmeraldas River Basin, Ecuador

**DOI:** 10.1371/journal.pone.0343879

**Published:** 2026-04-22

**Authors:** Kevin P. Chugá-Puetate, María Peñaranda-Valla, Daniel Escobar-Camacho, Jonathan Valdiviezo-Rivera, Juan F. Rivadeneira

**Affiliations:** 1 Carrera de Biología, Facultad de Ciencias Biológicas, Universidad Central del Ecuador, Numa Pompilio Llona and Yaguachi, Quito, Ecuador; 2 Fundación Cóndor Andino, Tamayo and Lizardo García, Quito, Ecuador; 3 Laboratorio de Ecología Acuática, Global Research & Solutions Center USFQ, Universidad San Francisco de Quito, Quito, Ecuador; 4 Instituto Nacional de Biodiversidad, Rumipamba and Shyris, Quito, Ecuador; Pontificia Universidade Catolica do Rio Grande do Sul, BRAZIL

## Abstract

*Astroblepus* is a genus of endemic Andean catfishes with problematic taxonomy because of cryptic diversity, ambiguous historical descriptions, and morphological plasticity. This study applied an integrative taxonomy approach—combining DNA barcoding (COI gene), geometric morphometrics, and traditional morphological characters—to assess the species diversity within the genus *Astroblepus* in the Esmeraldas River basin (Ecuador), where five species are currently recognized. A total of 395 specimens were analyzed (386 for morphometrics, 33 for genetics), integrating both new and publicly available sequences. The molecular analysis delimited seven evolutionary lineages, exceeding previously known diversity. The validity of *A. eigenmanni* and *A. fissidens* was confirmed; a possible synonym between *A. mindoensis* and *A. theresiae* was suggested, and *A.* aff. *mindoensis* was recovered as their sister group. Within *A. cyclopus* we identified two cryptic lineages (5.6% divergence), and two new lineages (*Astroblepus* sp. and *A.* aff. *mindoensis*) were discovered, characterized by distinct morphometric autapomorphies. Geometric morphometrics revealed four morphological clusters, with significant segregation between *A. cyclopus* and *Astroblepus* sp., but overlap within more complex groups. Altitudinal distribution and isolation among sub-basins may be drivers of divergence. These results reveal an underestimated diversity in the basin, highlighting the need for formal taxonomic revisions, sampling in unexplored areas, and urgent conservation strategies considering habitat fragmentation.

## Introduction

Astroblepidae is a monophyletic family within the order Siluriformes [1,2], represented solely by the genus *Astroblepus* (Humboldt 1805). Currently, there are 82 valid species [3], distributed from Panama to Bolivia, inhabiting elevations between 100 and 4000 m a.s.l. [4]. In Ecuador, 24 species occur, ranging from the western to the eastern slopes of the Andes, at elevations from around 300–3000 m a.s.l. [3,4]. They are commonly known as “climbing catfishes” or “preñadillas” [4] and possess unique morphological adaptations, such as a dorsal opening to the branchial chamber located between the dorsal margin of the operculum and the ventral edge of the pterotic bone, and a modified pelvic musculature that allows the alternate contraction of the muscles positioned anterior and posterior to the pelvic girdle, facilitating their movement upstream and even across vertical surfaces [5,6].

Astroblepids exhibit a restricted distribution, influenced primarily by altitude and temperature, with limited longitudinal movement, due to their range being confined to specific river stretches within each basin [4]. This restricted distribution prevents them from crossing adjacent stretches or other basins, thereby reinforcing their endemism and making them highly specialized in the habitats they occupy [4,7,8]. This endemism also rendered them particularly vulnerable to environmental changes, leading to population declines due to anthropogenic disturbances such as river course diversion, deforestation, overfishing, the use of harmful chemicals, and habitat fragmentation caused by the introduction of exotic species [9–12].

Research on the diversity and ecology of this group is limited [10,11], and its species level taxonomy is considered questionable and problematic [5,11,13], due to (1) high cryptic diversity [11], (2) morphological overlap among taxa [4], (3) inadequate original descriptions lacking critical documentation such as: (i) imprecise type localities (e.g., “Andes of Ecuador” for *A. fissidens*), (ii) use of few morphological characters to describe them, and (iii) no illustrations of specimen; it is worth noting that many of the 61 species were described before 1950 and lack these essential details [5,10,14]. Additionally, species in this genus exhibits high levels of inter and intraspecific variation and cryptic diversity, as evidenced by patterns of body morphology and coloration [10], which can lead to misidentification, resulting in either underestimation or overestimation of its diversity [5,11]. Therefore, research diversity and ecology are hampered by the fact that *Astroblepus* specimens in scientific collections are often misidentified, not identified, or assigned uncertain taxonomic status (e.g., “sp.,” “aff.,” or “cf.”) [14], highlighting the challenges in the taxonomy of this genus.

Ochoa et al. [11] conducted a molecular analysis of *Astroblepus* species from Colombia, Ecuador, Peru, and Bolivia, identifying a total of 25 well‐defined lineages, which include eight valid species and 17 possible new species. The results revealed taxonomic incongruences in the genus, uncovering potential synonymies, as well as the presence of cryptic species and phenotypic polymorphism [11,15]. Lawry [16] analyzed astroblepids from the Napo River basin in Ecuador and found that COI gene sequence did not account for morphological variation among 12 of the 14 morphospecies studied.

The study revealed cryptic diversity, suggesting that these morphospecies might group into only two distinct species. However, body coloration—a key criterion used to define the morphospecies—is a highly variable intraspecific trait [11], which could lead to misidentifying a single lineage as multiple morphospecies. This finding highlights the need to integrate quantitative methods to analyze morphological variation, such as geometric morphometrics, which has proven effective at capturing modular patterns in the evolution of complex characters in Siluriformes [17]. Recent studies in related Loricariidae groups suggest that geometric morphometrics, by preserving the spatial structure of characters, could more accurately quantify variation in attributes like dorsoventral compression or oral disc shape, thereby reducing subjectivity in morphological delimitations [18].

Ecuadorian samples included in the studies by Lawry [16] and Ochoa et al. [11] come from the Amazonian and coastal slopes of southern Ecuador, respectively. Consequently, molecular information is lacking for astroblepids from the western slope of northern Ecuador. The Esmeraldas River basin, in northwestern Ecuador, is ideal for studying *Astroblepus* due to its diversity, which includes at least five known species: *Astroblepus cyclopus* (Humboldt, 1805), *Astroblepus mindoensis* (Regan, 1916), *Astroblepus theresiae* (Steindachner, 1907), *Astroblepus eigenmanni* (Regan, 1904), and *Astroblepus fissidens* (Regan, 1904) [13]. These species were originally described based on meristic and traditional morphologic characters (e.g., body proportions) that exhibit interspecific overlap [19], underscoring the need for multivariate approaches to discern hidden morphological patterns [13]. The diversity and taxonomic complexity of astroblepids, along with contradictions in their classification, highlight the urgency of studying the Esmeraldas River basin. Furthermore, this region faces severe threats such as deforestation and pollution, which result from the expansion of agriculture and urban areas, as well as the construction of dams for irrigation and electricity generation—activities that are destroying essential habitats for these species [13,20]. The gaps in taxonomic knowledge, combined with human pressures, make this research fundamental not only to address taxonomic questions but also to understand their ecology and guide conservation strategies to protect their unique ecosystems [20].

In the present study, we applied an integrative taxonomic framework combining morphological analyses (discrete external characters), geometric morphometrics to capture shape variation quantitatively, and mitochondrial DNA sequences [17,18,21]. The mitochondrial dataset comprised both newly generated sequences produced for this study and additional sequences retrieved from public repositories, together creating a dataset of *Astroblepus* species from the western slope of Ecuador [10,11,15,22,23] and delimiting *Astroblepus* species in the Esmeraldas River basin using an integrative taxonomy approach by combining multiple lines of evidence to efficiently refine and define boundaries between species [24].

Based on current studies and available evidence, we hypothesize that the Esmeraldas River basin may harbor previously undescribed *Astroblepus* diversity that could exceed the five currently recognized species in the region. This study underscores the crucial importance of detailed investigations and exhaustive documentation to better understand and conserve the unique biodiversity of the Esmeraldas River basin.

## Materials and methods

### Study area

The Esmeraldas River basin, located in northwestern Ecuador, is the second largest in the country’s western slope. It covers approximately 21,640 km² and is fed by tributaries that flow through the provinces of Esmeraldas, Pichincha, Santo Domingo de los Tsáchilas, Manabí, and Cotopaxi. Its altitude varies from 3,516 m a.s.l. down to sea level, with average temperatures between 18 °C and 27 °C. The basin is characterized by the presence of montane and piedmont evergreen forests, but is also highly disturbed by illegal mining, deforestation, agricultural expansion, and dams which threaten its ecological integrity [13,19,20].

### Specimens analyzed and ethics declaration

Sequencing procedures complied with Ecuadorian legislation, specifically the Framework Agreement of the National Institute of Biodiversity (INABIO) for Access to Collection Permits and Genetic Resources (MAAE-DBI-CM 2021−0152) and the genetic resources access permit of the Universidad San Francisco de Quito (USFQ) (MAATE-DBI-CM-2023–0289). No additional field collections were conducted for this study; only specimens previously deposited in accredited scientific collections were analyzed. All procedures followed national and institutional ethical standards for the handling and use of biological material in Ecuador.

A total of 395 *Astroblepus* specimens deposited in INABIO, Museo de Historia Natural Gustavo Orcés V. (MEPN), and USFQ collections were examined (S1 Table). All voucher specimens used were already cataloged and deposited in the INABIO collection under reference numbers MECN 4948–4957 and 5007–5013, as well as in the MEPN collection under reference numbers MEPN 19719, 19722, 19725, 19727, 19731, 19741, 19755, and 20078. Of these 395 specimens, 33 had genetic sequences available: 21 were newly sequenced for this study, and 11 were reported previously (nine from Escobar-Camacho et al. [23]: eight *A*. aff. *mindoensis* and one *A. cyclopus*; plus, three *A. mindoensis* from Nirchio et al. [15]) (S2 Table). Only these museum specimens were analyzed.

### DNA extraction, PCR amplification, and sequencing

For 16 samples of *Astroblepus eigenmanni*, *A. fissidens*, *A. theresiae*, *A. mindoensis*, and *A. cyclopus*, DNA was extracted from tissue preserved in 96% ethanol using the commercial BioGENA kit. Polymerase chain reaction (PCR) was employed to amplify partial sequences of the cytochrome c oxidase subunit I (COI) gene using the primers FishF1 (5′-TCAACCAACCACAAAGACATTGGCAC-3′) and FishR1 (5′-TAGACTTCTGGGTGGCCAAAGAATCA-3′) [25]. These procedures—including extraction, amplification, and sequencing—were carried out by BioGENA (Quito-Ecuador). The other five sequences of *A. eigenmanni* (SP1–SP4) and one of *Astroblepus* sp. (TAN) were sequenced following the protocol described by Escobar-Camacho et al. [23].

### Species identification

**Fig 1:**
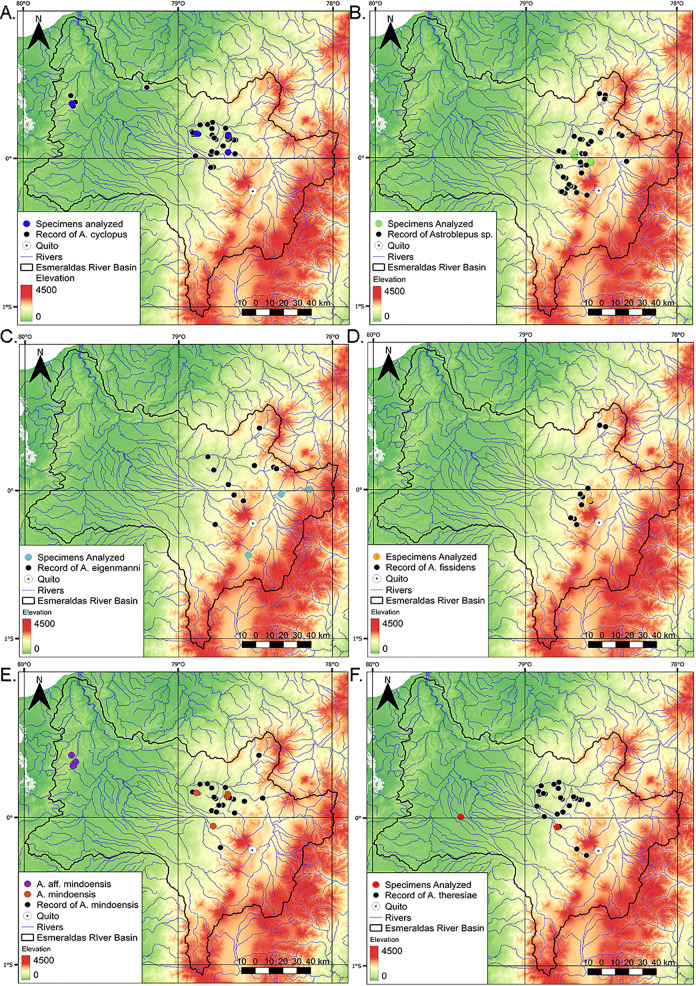
Maps of northern Ecuador illustrating the collection records of the samples used in this study. **A.** *A. cyclopus*; **B.** *Astroblepus* sp.; **C.** *A. eigenmanni*; **D.** *A. fissidens*; **E.** *A. mindoensis* / *A.* aff. *mindoensis*; and **F.** *A. theresiae*. The locations of specimens used in the genetic analysis are highlighted in colors corresponding to the clusters in the ABGD tree. Black indicates species distributions according to the INABIO database. Maps were prepared using layers from the IGM Geoportal [29], NASA/METI/AIST/Japan Spacesystems and U.S./Japan ASTER Science Team [30] and HydroSHED [31], processed with the QGIS software [32].

Taxonomic identification was based on original species descriptions reported from the basin [13,19], including *Astroblepus cyclopus* [26], *A. mindoensis* [27], *A. theresiae* [28], *A. eigenmanni*, and *A. fissidens* [19]. An additional morphotype that did not match any of these descriptions was provisionally designated as *Astroblepus* sp., with its records verified using the INABIO, MEPN, and USFQ databases (Fig 1).

Based on the observed characters, the taxonomic key was constructed. Three diagnostic external morphological characters following Regan [19]: premaxillary dentition, presence/absence of an adipose fin, and development of the adipose spine. Quantitative traits (maximum adipose-fin height and adipose-spine length) were calibrated from images with a 1 cm scale reference and direct measurements and expressed as a percentage of standard length (SL) to remove body size effects. Diagnostic thresholds (e.g., adipose fin < 2% SL vs. ≥ 3% SL) were defined from discontinuities in character distributions and optimized to maximize taxonomic separation. The key was assembled by ordering characters from highest to lowest discriminatory power and empirically validated using 395 specimens.

### Morphometric analysis

We analyzed 386 optimally preserved specimens (24 with genetic data), which were pinned in standard orientation and photographed (dorsal, lateral, ventral) using a Canon EOS 7D with 100 mm lens. Following Black & Armbruster [18], we digitized 20 dorsal, 19 lateral and 18 ventral landmarks (S1 Fig), adding only the adipose‐fin origin. Landmarks were digitized in TPSDig2 v2.32 [33] and Procrustes‐aligned in MorphoJ v1.08.02 [34], where we also generated group consensus shapes (S1–S3 Files). To remove allometry, we regressed shape on log10‐centroid size and used the residuals for a Canonical Variates Analysis in MorphoJ [17]; canonical scores and plots were exported to R [35]. Statistical support was assessed via Goodall’s F (10 000 permutations, p < 0.0001) [34]. Deformation grids and partial warps identified the anatomical points of greatest divergence, and consensus shapes for each species were generated using relative warps in tpsRelw32 [33] and the Thin-Plate Spline function [36] to infer morphological differences as landmark deformations on a grid calculated relative to each consensus configuration [33].

### Genetic analysis

The genetic analysis included 33 COI sequences of *Astroblepus* from the Esmeraldas basin, together with 128 sequences from Ochoa et al. [11], 32 from Schaefer et al. [10], and 25 from Jiménez-Segura et al. [22]. Three Loricariidae species (*Loricaria simillima* Regan, 1904; *Lamontichthys stibaros* Isbrücker & Nijssen, 1978; and *Pterygoplichthys multiradiatus* (Hancock, 1828)) were designated as the outgroup, since Loricariidae is the sister family to Astroblepidae [2,37]. All sequences are available in GenBank (S2–S4 Tables).

Sequences were edited in BioEdit v. 7.0.9 (Hall 1999) and aligned using the MAFFT algorithm [38]. For species delimitation, we employed the Automatic Barcode Gap Discovery method (ABGD), which estimates interspecific divergence relative to intraspecific variation [39]. ABGD was applied in previous *Astroblepus* studies [11] and in other taxa [40–43], demonstrating its efficacy in identifying species boundaries. In this analysis, we used a maximum prior intraspecific divergence (P max) of 0.15 and a relative gap width of 1.5, with the Jukes–Cantor (JC69) model and other default parameters established by Ochoa et al. [11], implemented via the ABGD web server (https://bioinfo.mnhn.fr/abi/public/abgd/abgdweb.html). These parameters help avoid lumping highly divergent species and facilitate detection of genetic discontinuities among species [39]. To calculate genetic distances among the groups identified by ABGD, we used MEGA 11 [44] with the JC69 model (S6 and S7 Tables). Additionally, the Assemble Species by Automatic Partitioning (ASAP) method was employed in iTaxoTools (http://galaxy.itaxotoolsweb.org/), using the JC69 substitution model and default parameters. ASAP generates species partitions ranked by a quality score without requiring prior assumptions about intraspecific diversity, providing an alternative perspective on species delimitation based on genetic distances [45]. We used the Jukes–Cantor (JC69) substitution model to compute genetic distances, this facilitates comparability with previous *Astroblepus* studies that employed JC69 [11] and, as a simple model, reduces the risk of overparameterization for single-marker analyses [46].

The reference phylogeny was reconstructed to infer evolutionary relationships among the studied groups. A maximum-likelihood (ML) tree was estimated in W-IQ-TREE (http://iqtree.cibiv.univie.ac.at) [47], using the TPM2u+F + I + G4 substitution model (LogL = –6145.3840) and 1 000 bootstrap replicates. The best-fit substitution model was automatically selected by IQ-TREE based on the Bayesian Information Criterion (BIC). Also, a ML distance matrix was computed with the phangorn package [48], and an initial topology was obtained using UPGMA. The resulting tree was converted to an ultrametric form using the ape package in R [35], without applying absolute calibrations. Topological support was further assessed in RAxML [49] through five searches initiated from randomized maximum-parsimony starting trees, employing 1,000 bootstrap replicates and the autoMRE stopping criterion.

Complementary species delimitation analyses included the bPTP model [50], which estimates the probability of speciation for each clade according to the observed branching patterns. The Bayesian implementation of PTP (bPTP) was run on the PTP online server (http://species.h-its.org), using the best ML tree obtained previously as input. The GMYC model [51,52] distinguishes between speciation and population coalescence processes by analyzing the distribution of node intervals in an ultrametric tree. A single-threshold GMYC model was applied using default parameters on the GMYC web server (http://species.h-its.org/gmyc/). This method detects temporal shifts between the Yule diversification process and within-species coalescent processes [52].

### Integrated analysis

We combined external morphological characteristics, morphometric, and molecular data for 24 specimens into a single matrix and inferred maximum-parsimony trees. The morphological characters were coded into a presence/absence matrix, while the morphometric landmark coordinates were treated as ordered continuous characters [53], and the molecular data were included as an aligned nucleotide matrix. All tree searches were carried out in TNT v.1.6 [54]; heuristic searches used ten random addition sequences, TBR branch swapping in 30 replicates, and ten trees saved per replicate [55]. Clade support was assessed using the Bremer support index, which estimates the stability of clades to topological perturbations [56]. Trees were rooted on *Loricaria simillima* and final trees were edited in FigTree v1.4.4 [57].

## Results

**Fig 2:**
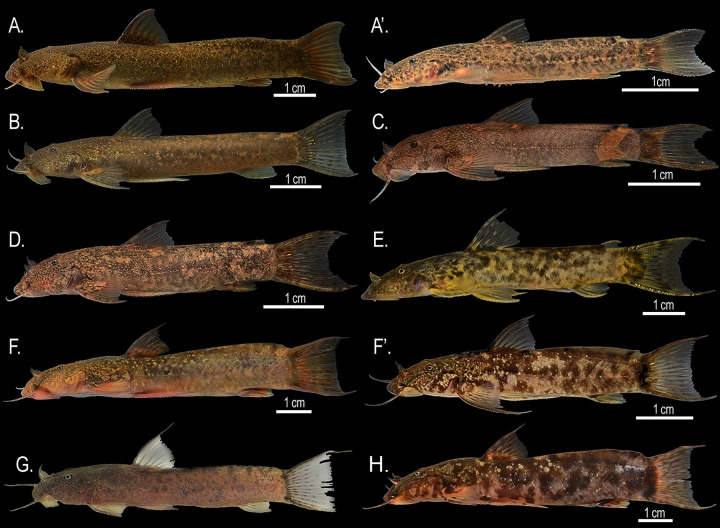
Species of *Astroblepus* from the Esmeraldas River basin. **A.** *A.eigenmanni* (adult), A′. *A. eigenmanni* (juvenile), **B.** *A. fissidens*, **C.** *Astroblepus* sp., **D.** *A. cyclopus* (Alambi River), **E.** *A. cyclopus* (Mashpi River), **F.** *A. mindoensis* (Alambi River), F′. *A. mindoensis* (Mindo River), **G.** *A.* aff. *mindoensis* (Cube River), and **H.** *A.s theresiae* (Mashpi River). Pictures courtesy of Jaime Culebras **(B)**, Karla Barragán **(G)**, and Juan Francisco Rivadeneira (remaining images).

The initial morphological analysis of the 395 specimens enabled their identification and separation into six taxonomic categories: *Astroblepus cyclopus* (n = 123), *Astroblepus* sp*.* (n = 117), *A. eigenmanni* (n = 53), *A. theresiae* (n = 30), *A. fissidens* (n = 16), and *A. mindoensis* (n = 47), the latter including specimens of *A.* aff*. mindoensis* (n = 8) (Fig 2). To differentiate between *Astroblepus* species from the Esmeraldas River basin, the following dichotomous key was applied:

**Table:** 

**1.** Adipose-fin, maximum height < 2% standard length............................	2.
**1’.** Adipose-fin, maximum height ≥ 2% standard length............................	3.
**2.** Adipose-spine ≥ 10% anteroposterior length of adipose-fin; spine extending beyond posterior margin of anal-fin (Figs 2D and 2E)............................	*A. cyclopus*
**2’.** Adipose-spine < 10% anteroposterior length of adipose-fin; spine not extending beyond posterior margin of anal-fin (Fig 2C)............................	*Astroblepus* sp.
**3.** External premaxillary teeth not all bicuspid; four central teeth bicuspid, remaining teeth unicuspid............................	4
**3’.** External premaxillary teeth all bicuspid (Fig 2B)............................	*A. fissidens.*
**4.** Adipose-fin continuous with caudal-fin base; juveniles without adipose-spine............................	5.
**4’.** Adipose-fin separated from caudal-fin base; juveniles with minute adipose-spine (< 10% anteroposterior length of adipose-fin) (Fig 2A′)............................	*A. eigenmanni*.
**5.** Adipose-spine externally visible; projecting from dorsal margin of adipose-fin (Fig 2F, 2F′ and 2G)............................	*A.* aff*. mindoensis* and *A. mindoensis*.
**5’.** Adipose-spine not externally visible; completely embedded in adipose-fin base (Fig 2H)............................	*A. theresiae*.

This preliminary morphological characterization established reference identities underpinning the subsequent morphometric, genetic, and comprehensive integrated analyses.

### Geometric morphometrics analysis

**Fig 3:**
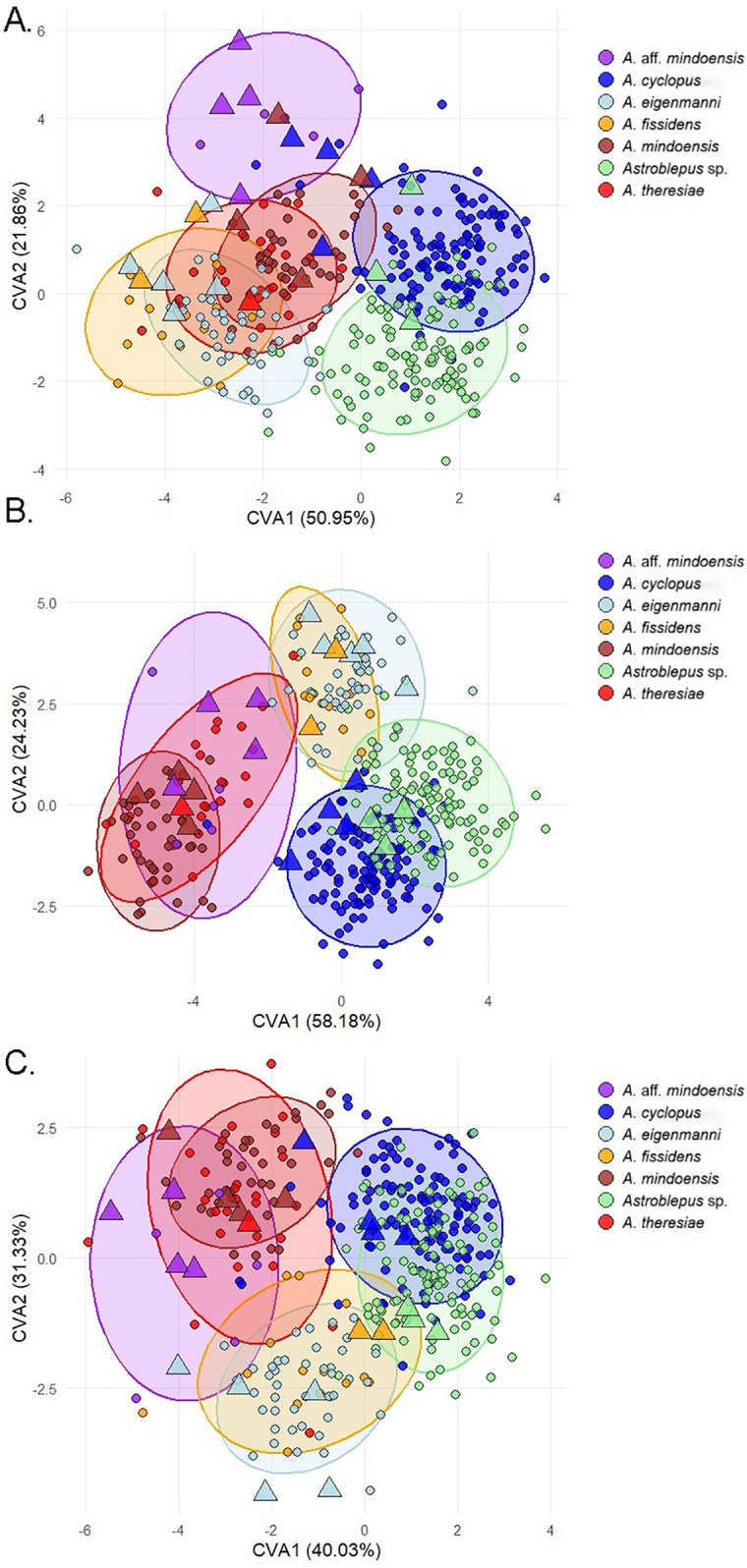
Canonical Variate Analysis (CVA) with allometry correction for seven *Astroblepus* species: (A) dorsal view, (B) lateral view, and (C) ventral view. Triangles denote specimens with genetic sequences, circles denote other specimens, and each color corresponds to a different species. Ellipses represent 95% confidence intervals.

Size-corrected CVA revealed four discrete clusters across all species (Fig 3). *Astroblepus cyclopus* and *Astroblepus* sp. were spatially segregated with minimal overlap, whereas the other species formed two overlapping clusters: (1) *A. mindoensis, A. theresiae*, *A*. aff. *mindoensis* and (2) *A. eigenmanni*, *A. fissidens.* Permutation tests on Procrustes distances confirmed significant divergence between *A. cyclopus* and *Astroblepus* sp. (p < 0.0001), but not within the other two clusters (S5 Table).

The most significant morphological landmarks among the consensus configurations in dorsal, lateral, and ventral views corresponded to oral morphology, adipose-fin position, pectoral-fin insertion, and overall body conformation (S2 Fig). *Astroblepus cyclopus* was characterized by a broad body, reduced oral disk, and centrally positioned adipose fin relative to the body axis (S2 Fig). In contrast, *Astroblepus* sp*.* exhibits marked anteroposterior flattening, a small mouth, broad oral disk, and an adipose fin origin displaced toward the caudal region (S2 Fig).

*Astroblepus eigenmanni* and *A. fissidens* share similar fin arrangements: the adipose fin originates near the posterior end of the dorsal fin, and the pectoral fins insert at the midpoint level of the opercle. Their primary divergence lies in body conformation: *A. eigenmanni* displays an elongated body with slight lateral compression, whereas *A. fissidens* exhibit greater depression (S2 Fig). *Astroblepus mindoensis* and *A. theresiae* possess a robust body with bulging at the dorsal-fin origin and adipose-fin insertion posterior to the termination of the latter, lacking distinctive features (S2 Fig). Finally, *A.* aff. *mindoensis* possesses a robust and elongated body, a moderately compressed head, pectoral-fin insertion below the opercle midpoint, and the mouth opening sufficiently broad to cover nearly the entire oral disk (S2 Fig).

### Genetic analysis

A total of 221 COI gene sequences (682 bp) from *Astroblepus* were analyzed. No insertions, deletions, stop codons, or sequencing errors were detected. Nucleotide composition was: 26.39% adenine, 26.84% cytosine, 17.2% guanine, and 29.57% thymine. Transitions (79.97%) were more frequent than transversions (20.02%). The multiple sequence alignment identified 408 conserved sites, 274 polymorphic sites, and 226 parsimony-informative sites.

Species delimitation within the genus *Astroblepus* using the ABGD and ASAP analysis converged on the same set of 35 genetic lineages. In contrast, bPTP and GMYC identified 147 and 75 lineages, respectively, showing greater fragmentation in the phylogeny-based approaches. In the Esmeraldas Basin dataset, *A. cyclopus* was consistently delimited as an independent lineage across all analyses. *Astroblepus eigenmanni* was recovered as a single lineage under ABGD but was either split or grouped with other species in the remaining methods. *Astroblepus fissidens* was divided into two lineages in bPTP and GMYC. Populations assigned to *A.* aff*. mindoensis* and the *A. theresiae*/*mindoensis* complex showed discrepancies: ABGD and ASAP grouped them into a single lineage, whereas GMYC and bPTP subdivided them, suggesting potential cryptic diversity. Similarly, the undescribed entity *Astroblepus* sp*.* was confirmed as an independent lineage in nearly all analyses, except GMYC, which divided it into two sublineages (Table 1).

**Table 1:** Mean interspecific genetic divergence (JC69) of lineages of Esmeraldas basin. Standard deviations are shown in the upper triangle of the matrix.

Lineages	*A. theresiae /mindoensis*	*A*. aff. *mindoensis*	*A. eigenmanni*	*Astroblepus* sp.	*A. cyclopus* Cube	*A. cyclopus* Alambi	*A. fissidens*
***A. theresiae /mindoensis***		0.014	0.015	0.017	0.019	0.016	0.016
***A.* aff. *mindoensis***	0.071		0.017	0.016	0.019	0.015	0.015
***A. eigenmanni***	0.084	0.094		0.009	0.013	0.009	0.017
***Astroblepus* sp.**	0.089	0.082	0.033		0.014	0.010	0.019
***A. cyclopus* Cube**	0.115	0.115	0.052	0.063		0.013	0.019
***A. cyclopus* Alambi**	0.078	0.081	0.032	0.031	0.056		0.017
***A. fissidens***	0.084	0.076	0.090	0.103	0.118	0.089	

**Fig 4:**
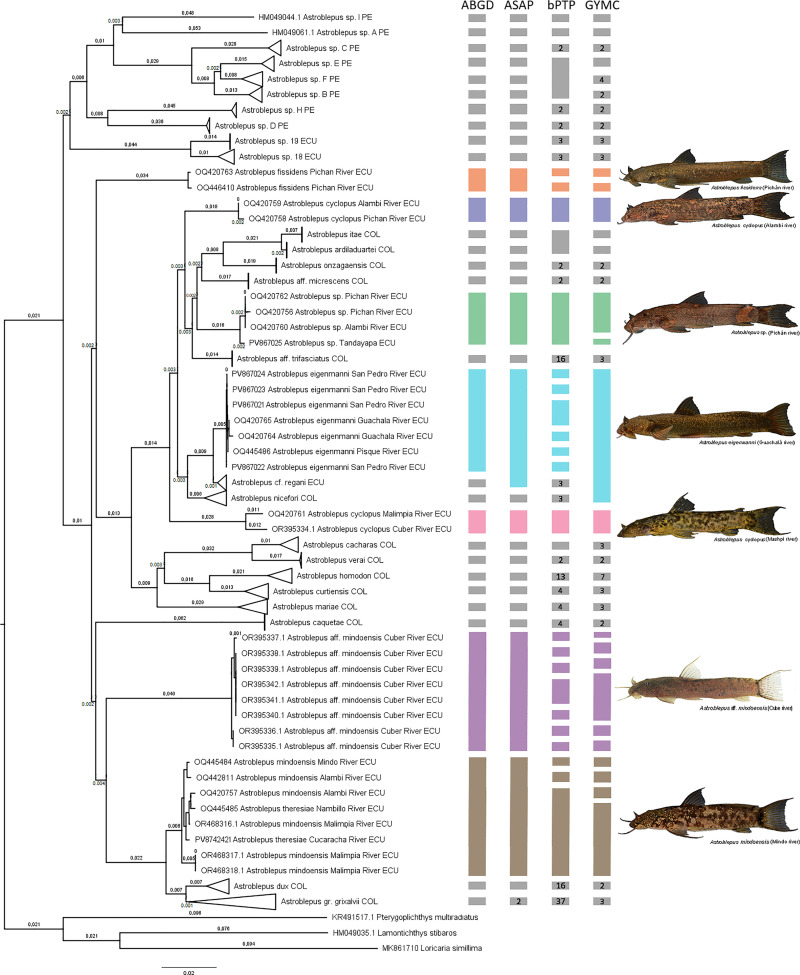
Tree resulting from the ABGD, ASAP, bPTP, and GMYC analyses based on the cytochrome c oxidase subunit I (COI) gene. The colored bars next to terminals represent distinct *Astroblepus* lineages from the Esmeraldas Basin, while lineages from other regions are indicated in gray. Genetic distances are shown above each branch. GenBank accession numbers preceded species names, except in collapsed branches. The river of origin and corresponding country follow each species name; species outside the Esmeraldas Basin are labeled with country only. Abbreviations: PE (Peru), COL (Colombia), ECU (Ecuador).

Overall, ABGD and ASAP exhibited high concordance (35 lineages), while GMYC and bPTP produced more fragmented delimitations. The remaining lineages correspond to species delimited by Ochoa et al. [11], Schaefer et al. [10], and Jiménez-Segura et al. [22], while the final three species represent the outgroup (Fig 4).

Mean interspecific genetic distance to ABGD was 0.103 ± 0.031, ranging from 0.161 ± 0.024 between *Astroblepus verai* and *Astroblepus sp. H*, to 0.006 ± 0.004 between *A. itae* and *A. ardiladuartei* (S6 Table). The topology of the tree showed *A. theresiae* nested within *A. mindoensis,* and the low pairwise genetic distance between them (0.008 ± 0.003; < 2%), further supported that they should not be considered distinct species. In contrast, *A*. *theresiae/mindoensis* and *A*. aff. *mindoensis* diverged by 0.071 ± 0.014—despite prior assumptions of their close affinity—indicating a clear genetic separation. The topology of the tree also resolved the two *A. cyclopus* groups as well-supported, reciprocally monophyletic clades; their genetic distances (0.056 ± 0.012; > 2%), reinforces their status as separate lineages. Other lineages showed an average intraspecific distance of 0.003 ± 0.001, with no lineage exceeding genetic distance threshold (S7 Table).

**Fig 5:**
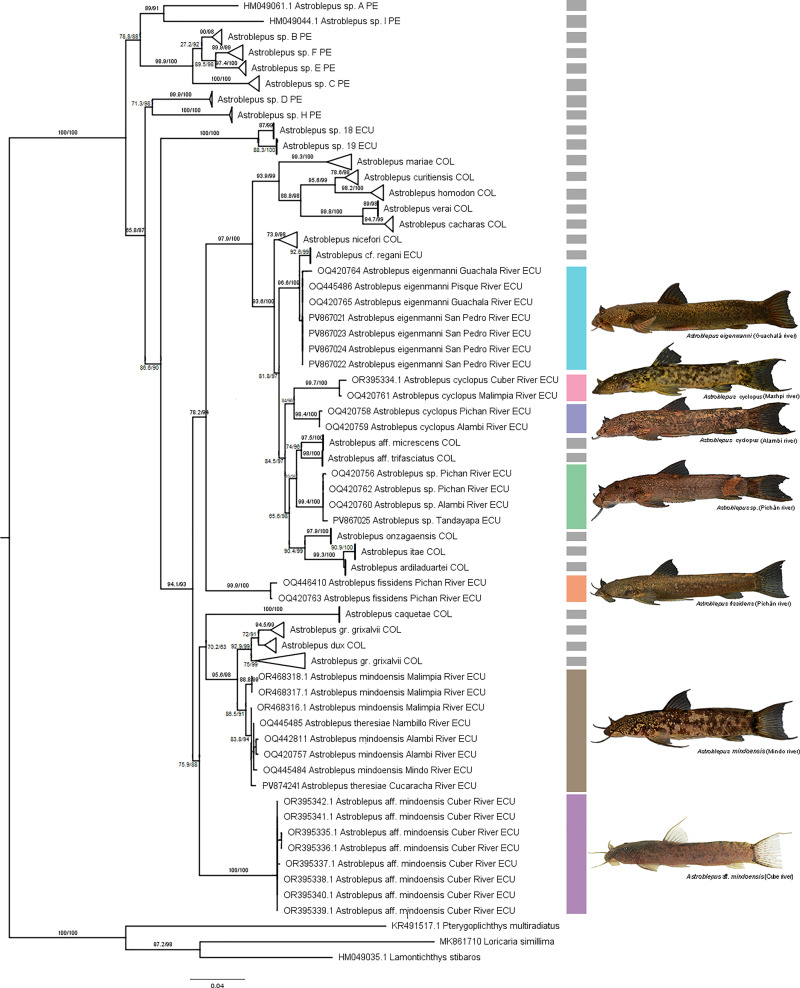
Maximum likelihood phylogenetic tree of *Astroblepus* based on the cytochrome c oxidase subunit I (COI) gene. The colored bars next to terminals represent each *Astroblepus* clade from the Esmeraldas Basin, while other species are shown in gray. SH-aLRT and Bootstrap values are indicated at nodes. GenBank accession numbers precede species names, except for collapsed branches. The river of origin and corresponding country follow each species name; species outside the Esmeraldas Basin include country designation only. Abbreviations: PE (Peru), COL (Colombia), ECU (Ecuador).

In the maximum likelihood analysis, *Astroblepus* was recovered as a monophyletic group. Of the thirty-five lineages identified, thirty were represented as monophyletic groups, two were represented by single individuals, and three belonged to the outgroup. The analysis revealed that species from the Esmeraldas River basin were not recovered as a monophyletic assemblage; instead, they form sister groups to Colombian lineages. Specifically, the *A.* aff. *mindoensis*, *A. theresiae*, and *A. mindoensis* clades were resolved as sister to *A*stroblepus *grixalvii* group. The *A. cyclopus* clades were recovered as a monophyletic group. Furthermore, the analysis indicated that other lineages – *A. eigenmanni*, *A. fissidens*, and *Astroblepus* sp*.* – formed distinct, independent clades, demonstrating their unique evolutionary trajectories within the broader phylogeny (Fig 5).

### Integrated analysis

**Fig 6:**
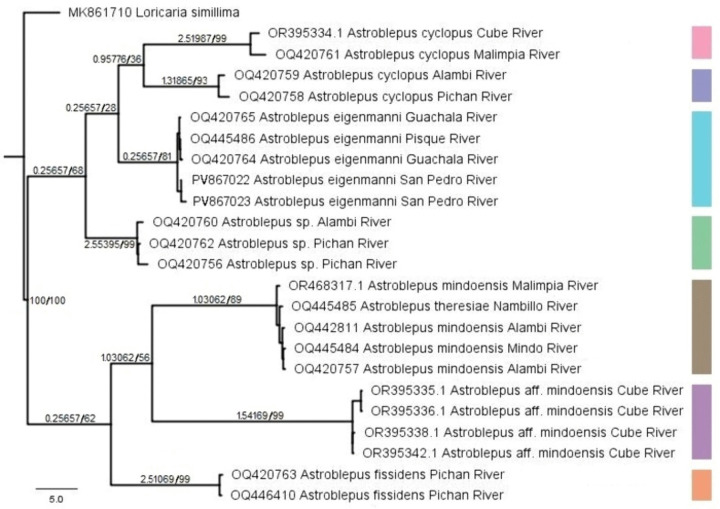
Tree inferred under the Maximum Parsimony (MP) criterion based on morphological, morphometric, and genetic character matrices, corresponding to the seven *Astroblepus* morphospecies. Bremer support and bootstrap values are indicated above main branches (left) along with resampled values (right).

The Maximum Parsimony (MP) analysis of all data recovered seven clades corresponding to the analyzed taxonomic lineages. Topologies derived from combined analyses showed structural congruence, with medium to high nodal support values (Fig 6) on all branches associated with major groups. The integrated data matrix produced the most parsimonious tree (minimum length = 330.863; consistency index = 0.301). In the consensus topology, *Astroblepus cyclopus* resolved into two monophyletic sisters groups. *Astroblepus fissidens* formed an independent lineage nested within a clade containing *A. mindoensis*, *A. theresiae*, and a separate *A.* aff*. mindoensis* clade. Concurrently, *Astroblepus* sp*.* and *A. eigenmanni* were recovered as well-supported monophyletic groups.

## Discussion

Our integrative study revealed undescribed cryptic diversity within *Astroblepus* in the Esmeraldas Basin, where the convergence of morphological, morphometric, and genetic data delineated seven taxonomic lineages. Morphological diagnosis recovered six lineages—five corresponding to described species (*A. cyclopus*, *A. mindoensis*, *A. eigenmanni*, *A. theresiae* and *A. fissidens*) plus one unassigned morphotype (*Astroblepus* sp.) (Fig 2). Geometric morphometrics then identified four discrete clusters: *A. cyclopus* and *Astroblepus* sp. were spatially segregated, *A. mindoensis* clustered with *A. theresiae* and *A*. aff. *mindoensis*, and *A. eigenmanni* grouped with *A. fissidens*, the last named pair differing primarily in body compression. This discordance between classical methods and genetic delimitation aligned with prior warnings about taxonomic complexity in Andean fishes [10,11], suggesting limitations of traditional approaches in capturing cryptic diversity.

Maximum parsimony analyses corroborated this complexity by identifying seven well-defined genetic lineages, exceeding previous estimates. Two lineages unambiguously corresponded to *A. eigenmanni* and *A. fissidens*, while four exhibited uncertainties: two lineages within *A. cyclopus*, one grouping *A.* aff. *mindoensis*, and another comprising the *A. mindoensis*–*A. theresiae* duo. The seventh lineage (*Astroblepus* sp.), along with the *A*. aff. *mindoensis* likely represents a distinct, undescribed species, each supported by both morphometric and genetic characters. The results of the ASAP, bPTP, and GMYC analyses recovered a slightly higher number of lineages than previously estimated; however, this difference may reflect the sensitivity of these algorithms to intrinsic variation in COI polymorphism (e.g., overlap between intra- and interspecific divergence, introgression, among others). For instance, GMYC may fail to assign entities in up to 20% of cases, which highlights the need for complementary data [58]. In contrast, the results of the ABGD and Maximum Likelihood analyses were consistent with the integrative Maximum Parsimony analysis, which recovered all seven clades with robust nodal support. We agree with Jiménez-Prado et al. [13] regarding the species count in the basin. However, we differ in several specific aspects: (1) the broad distribution attributed by the authors to *A. cyclopu*s; (2) the incorrect record of *A. grixalvii* and *A. longifilis* for the Esmeraldas basin; and (3) the absence of *A. whymperi* (Boulenger, 1890) in our findings. This latter point could be explained by sampling gaps in the southern portion of the basin, unrecognized distributional boundaries, or even local population declines.

Discrepancies in lineages attributed to *A. cyclopus* and the *A. mindoensis*/*A. theresiae*/*A.* aff*. mindoensis* complex reinforce Ochoa et al. [11] hypothesis of molecular underrepresentation in the genus, necessitating urgent taxonomic revision to reconcile morphological and genetic identities. This need extends to the biogeographic context, where the phylogeny revealed a disjunct pattern: sympatric species (e.g., *A. eigenmanni* and *A. fissidens*) did not share recent ancestry, with Esmeraldas basin lineages nested within Colombian clades. Taken together, these phylogenetic and geographic patterns indicate that geographic proximity does not determine evolutionary affinity, supporting historical dispersal events and vicariance as drivers of distribution in the Northern Andes [10,11,14]. Our results corroborate Nirchio et al. [15] and Ochoa et al. [11], positioning Ecuadorian lineages within the Nor-Andean clade, a dynamic biogeographic unit influenced by recent geological activity, explaining the observed complexity in speciation patterns.

### Delimitation of valid *Astroblepus* species

Our integrative approach corroborates the presence of *A. eigenmanni* and *A. fissidens* in the Esmeraldas Basin through a combination of morphological, genetic, and morphometric evidence. Diagnostic traits [19] were consistent with species-level identification, and DNA barcoding revealed >2% interspecific divergence, exceeding the heuristic threshold for species delimitation in teleost fishes [59]. For *A. eigenmanni*, specimens from the type localities were included in the analyses, providing a direct link to the original description. In contrast, the type locality of *A. fissidens* is imprecisely recorded as “Andes of Ecuador” [19], which introduce uncertainties that should be considered in future sampling efforts.

Consensus shape reconstruction revealed patterns of morphological convergence between the two species, particularly in fin arrangements, where they overlap in CVA. This homoplasy likely from shared selective pressures in Andean river habitats, such as substrate variability, turbulence, and oxygen gradients [60], which promote parallel adaptations in anatomically interdependent traits [18]. Such similar evolutionary trajectories explain the limitations of geometric morphometrics in delimiting sympatric species, as functional characters mask genetic divergence.

Phylogenetic analyses resolved this apparent contradiction by recovering both taxa as well-supported clades, demonstrating that their evolutionary histories transcend purely morphological signals. As proposed by Rabosky et al. [61], these decoupling underscores that speciation and morphological evolution may operate under phylogenetic constraints, and that only integrative approaches can reveal hidden divergence patterns. These findings emphasize the necessity of combining multiple lines of evidence in taxa with adaptive plasticity, particularly in complex ecosystems such as Andean streams.

### The *Astroblepus theresiae*/*mindoensis* complex

Our molecular analysis reveals that *A. mindoensis* and *A. theresiae* exhibit low genetic differentiation [59]. This minimal divergence is reflected in the presence of individuals with mixed morphological traits within both genetic lineages, challenging the validity of their taxonomic separation. Canonical Variates Analysis (CVA) and consensus shapes corroborates this lack of differentiation, showing continuous patterns in body shape resulting in complete overlap. Among all traits evaluated, only the adipose spine showed consistent differences: exposed in *A. mindoensis* versus embedded in *A. theresiae*. However, this character exhibits high intraspecific variation and lacks phylogenetic support [11]. These findings suggest that variations in the adipose spine may reflect phenotypic plasticity influenced by local environmental factors rather than evolutionary differentiation [15].

The taxonomic complexity is heightened by nomenclatural considerations: while we include samples of *A. mindoensis* from its type locality (“Río Mindo”) [27], the precise location of the type locality of *A. theresiae* (“Cayendelet”) remains unknown [28], precluding direct comparisons between topotypes. Additionally, although studies such as Nirchio et al. [15] provide detailed characterizations of *A. mindoensis*, the absence of comparison with *A. theresiae* and the grouping of their specimens within our shared lineage raises questions about the exclusivity of their identifications. This situation fits within a broader pattern where numerous nominal *Astroblepus* species show uncertain boundaries, particularly when considering their phylogenetic relationship with the *A. grixalvii* complex – a sister group exhibiting notable morpho-skeletal variations that may extend to this clade [62].

In contrast to this scenario, *A.* aff. *mindoensis* emerges as a clearly differentiated lineage, showing significant genetic divergence from the *A. theresiae/mindoensis* group, it possesses consistent diagnostic traits. Phylogenetically, this lineage forms part of the *‘A. grixalvii* complex alongside *A. mindoensis* and *A. theresiae*, but as an independent clade, supporting previous studies identifying cryptic species within this complex [11,15]. The combination of genetic distance (≥2%) and stable morphometric characters reinforces the possibility of representing distinct species within the complex.

These integrated results underscore the need for a comprehensive taxonomic revision that: 1) evaluates the potential synonym between *A. mindoensis* and *A. theresiae* considering the inconsistency of the adipose spine as a diagnostic character, 2) formally validates the status of *A.* aff*. mindoensis* as a new species, and 3) explores patterns of morphological variation within a phylogenetic context, particularly in relation to the *A. grixalvii* complex. Future studies should prioritize locating critical topotypes and conducting comparative analyses integrating ontogeny and ecological factors to resolve species boundaries within this morphologically conserved but genetically diverse group.

### The case of *Astroblepus cyclopus*: cryptic speciation in progress

Our results confirm that the two genetic lineages of *A. cyclopus* exhibit morphological characteristics consistent with the species’ original description [26]. However, the imprecise type locality (“Reino de Quito” [26]– a historically ambiguous region encompassing parts of Colombia, Ecuador, Peru, and Brazil; [63]) prevents assigning these lineages to the original type material although, Humboldt mentions volcanoes that are currently located in Ecuadorian Andes, specifically in the Esmeraldas Basin. This highlights the taxonomic challenges arising from historical descriptions with insufficient geographical data [14], particularly given that the original holotype is unknown and the four syntypes are missing [64], and underscores the critical need to designate neotypes with exact provenances in modern taxonomic revisions.

The marked genetic divergence between the lineages, coupled with their distribution in the Alambi and Cube rivers (Esmeraldas basin), suggests a scenario of incipient speciation mediated by geographic isolation. This partially contradicts the paradigm of *Astroblepus* being restricted to single or adjacent rivers [10], as both lineages coexist within the same basin but in distinct sub-basins, where a physical barrier may be driving their differentiation. Additional records of morphotypes attributed to *A. cyclopus* in Colombian basins [65,66] and Ecuadorian basins like the Guayas [13] reinforce the hypothesis that this taxon represents an undescribed cryptic species complex, geographically widespread but with fragmented population structures.

The absence of morphometric discontinuity between the lineages – despite their clear genetic segregation – points to potential cryptic speciation where morphological and molecular evolution are decoupled. Specimens from the *A. cyclopus* lineage in the Alambi River and the other lineage in the Cube River show continuous morphological variation that does not correlate with the two well-defined genetic lineages. This indicates that phenotypic changes may be lagging genomic divergence. This pattern aligns with “grey zone” speciation models [67], where morphology remains conserved in early stages of divergence while molecular markers accumulate differences, as documented in other Loricariidae like *Schizolecis guntheri* (Miranda Ribeiro, 1918) [68]. Disparate evolution among traits – where selection on adaptive characters (e.g., body shape in response to micro-environmental conditions) may be slower than neutral genetic drift [69] – would explain this apparent contradiction. Therefore, integrating ecological and behavioral data will be crucial to determine whether these lineages represent independent biological entities or a single taxon with complex genetic structure.

These integrated findings demand an urgent taxonomic revision that will: 1) designate neotypes with precise localities to stabilize the identity of *A. cyclopus*, resolving the ambiguity of “Reino de Quito”; According to the *Check List of the Freshwater Fishes of South and Central America* [64], no holotype is known for this species, and the four syntypes are missing. The whereabouts of the original type material remain uncertain, and no confirmed specimens appear to exist in collections. This absence of type material satisfies the conditions for the designation of neotypes, which would provide a well-documented reference with precise provenance; 2) evaluate the specific status of both genetic lineages (Alambi/Cube) using subtle diagnostic morphological characters or complementary biomarkers; and 3) explore the historical connectivity between populations in the Esmeraldas basin and Colombian/Guayas records to determine if they represent a cryptic species complex. Future studies must seek to discern whether the observed continuous variation reflects phenotypic plasticity or early stages of speciation within this fragmented river system.

## Conclusions

Integrating traditional morphology, geometric morphometrics, and COI DNA barcoding, we uncovered seven evolutionarily distinct *Astroblepus* lineages in the Esmeraldas Basin —surpassing the five species previously recognized and remarkably revealing cryptic diversity. This integrative framework validated *A. eigenmanni* and *A. fissidens* as discrete species, proposed the synonymy of *A. mindoensis* and *A. theresiae* based on minimal genetic divergence and continuous morphometric overlap (thereby invalidating the adipose spine as a diagnostic trait for these species), and identified two novel candidate taxa—*Astroblepus* sp. and *A*. aff. *mindoensis*—each supported by unique morphometric autapomorphies and significant genetic divergence. Furthermore, we documented two cryptic lineages within *A. cyclopus* that lack discernible morphological differentiation.

Given the extreme vulnerability of these endemics to habitat fragmentation and anthropogenic disturbance, our results underscore the urgent need for formal taxonomic revision, intensive sampling in understudied regions to delineate true distributions, and targeted conservation strategies tailored to the Andean topography that structures their gene flow. This will allow for an updated threat status for these species. Ultimately, this study demonstrates that only through comprehensive integrative approaches can we fully unravel the evolutionary complexity of this emblematic Andean genus.

## Supporting information

S1 Fig Landmark scheme for *Astroblepus* (dorsal, left lateral, and ventral views). (TIF)

S2 Fig Consensus body shape in dorsal, lateral, and ventral views. (TIF)

S1 Table Catalog number of all specimens analyzed. (XLSX)

S2 Table Voucher, tissue number, GenBank accession numbers, identification and geographic information of the analyzed samples from the Esmeraldas River basin. (DOCX)

S3 Table GenBank sequences of *Astroblepus* used in this study. (DOCX)

S4 Table GenBank accession numbers of the out group. (DOCX)

S5 Table Procrustes distances between pairs of *Astroblepus* species. P-values are shown in blue; values below 0.0001 indicate statistically significant differences.
(XLSX)

S6 Table Interspecific genetic divergence (JC69) of lineages. The standard deviation is shown in blue.
(XLSX)

S7 Table Intraspecific genetic divergence (JC69) of lineages. (XLSX)

S1 File Dorsal view morphometric data. (TXT)

S2 File Lateral view morphometric data. (TXT)

S3 File Ventral view morphometric data. (TXT)
