# Supplementary material for: Cryptic diversity in Astroblepus (Siluriformes: Astroblepidae): Integrative taxonomy reveals evolutionary complexity in the Esmeraldas River Basin, Ecuador
Source: PLoS One. 2026 Apr 22;21(4):e0343879. doi: 10.1371/journal.pone.0343879 (PMC13102232; doi:10.1371/journal.pone.0343879)
Supplement: S2 Table — (DOCX) [file pone.0343879.s004.docx]

**S4 Table.** Voucher, tissue number, GenBank accession numbers, identification and geographic information of the analyzed samples from the Esmeraldas River basin

| **Tissue #** | **Voucher Catalog #** | **Taxon** | **GenBank accession #** | **Coordinates** | **Locality** | **Altitude** | **References** |
| --- | --- | --- | --- | --- | --- | --- | --- |
| Cube62 | MEPN 19719 | *Astroblepus* aff. *mindoensis* | OR395342.1 | 0.37805N -79.66460W | Cuber River | 507 | Escobar-Camacho et. al. [1] |
| Cube61 | MEPN 19722 | *Astroblepus* aff. *mindoensis* | OR395341.1 | 0.37805N -79.66460W | Cuber River | 507 | Escobar-Camacho et. al. [1] |
| Cube3 | MEPN 19725 | *Astroblepus* aff. *mindoensis* | OR395340.1 | 0.351N -79.683W | Cuber River | 518 | Escobar-Camacho et. al. [1] |
| Cube2 | MEPN 19741 | *Astroblepus* aff. *mindoensis* | OR395339.1 | 0.351N -79.683W | Cuber River | 518 | Escobar-Camacho et. al. [1] |
| Cube45 | MEPN 19727 | *Astroblepus* aff. *mindoensis* | OR395338.1 | 0.35192N -79.68103W | Cuber River | 512 | Escobar-Camacho et. al. [1] |
| Cube52 | MEPN 19755 | *Astroblepus* aff. *mindoensis* | OR395337.1 | 0.35124N -79.67809W | Cuber River | 464 | Escobar-Camacho et. al. [1] |
| Cube23 | MEPN 19731 | *Astroblepus* aff. *mindoensis* | OR395336.1 | 0.423N -79.693W | Cuber River | 376 | Escobar-Camacho et. al. [1] |
| Cube22 | MEPN 20078 | *Astroblepus* aff. *mindoensis* | OR395335.1 | 0.423N -79.693W | Cuber River | 376 | Escobar-Camacho et. al. [1] |
| 20220818002AE1 | MECN 4955 | *Astroblepus eigenmanni* | OQ420764 | 0.00852N -78.15122W | Guachalá River | 1989 | This study |
| 20220818002AE3 | MECN 4955 | *Astroblepus eigenmanni* | OQ420765 | 0.00852N -78.15122W | Guachalá River | 1989 | This study |
| 20220818001AV1 | MECN 5007 | *Astroblepus eigenmanni* | OQ445486 | -0.02528S -78.33386W | Pisque River | 1990 | This study |
| SP1 | - | *Astroblepus eigenmanni* | PV867021 | -0.43553S -78.547747W | San Pedro River | 2708 | This study |
| SP2 | - | *Astroblepus eigenmanni* | PV867024 | -0.43553S -78.547747W | San Pedro River | 2708 | This study |
| SP3 | USFQ001 | *Astroblepus eigenmanni* | PV867022 | -0.43553S -78.547747W | San Pedro River | 2708 | This study |
| SP4 | USFQ002 | *Astroblepus eigenmanni* | PV867023 | -0.43553S -78.547747W | San Pedro River | 2708 | This study |
| 20211216001AF7 | MECN 4949 | *Astroblepus fissidens* | OQ446410 | -0.07037S -78.57886W | Pichán River | 2645 | This study |
| 20211216001AF4 | MECN 4949 | *Astroblepus fissidens* | OQ420763 | -0.07037S -78.57886W | Pichán River | 2645 | This study |
| Cube6 | - | *Astroblepus cyclopus* | OR395334.1 | 0.36833N -79.68583W | Cuber River | 526 | Escobar-Camacho et. al. [1] |
| 20210902AC1 | MECN 4951 | *Astroblepus cyclopus* | OQ420758 | 0.15736N -78.67671W | Pichán River | 1109 | This study |
| 2022021517AC1 | MECN 4948 | *Astroblepus cyclopus* | OQ420761 | 0.16647N -78.87937W | Malimpia River | 722 | This study |
| 20210908001AC1 | MECN 4950 | *Astroblepus cyclopus* | OQ420759 | 0.04078 N -78.67732 W | Alambi River | 1420 | This study |
| 20210902AW1 | MECN 4952 | *Astroblepus mindoensis* | OQ420757 | 0.15736N -78.67671W | Alambi River | 1109 | This study |
| 20220804AM2 | MECN 5012 | *Astroblepus mindoensis* | OQ442811 | 0.14394N -78.681403W | Alambi River | 1104 | This study |
| 20220825001AM1 | MECN 5008 | *Astroblepus mindoensis* | OQ445484 | -0.05857S -78.77261W | Mindo River | 1228 | This study |
| 436 | MECN 4956 | *Astroblepus mindoensis* | OR468318.1 | 0.16647N -78.87937W | Malimpia River | 722 | Nirchio et al. [2] |
| 432 | MECN 4956 | *Astroblepus mindoensis* | OR468317.1 | 0.16647N -78.87937W | Malimpia River | 722 | Nirchio et al. [2] |
| 431 | MECN 4956 | *Astroblepus mindoensis* | OR468316.1 | 0.16647N -78.87937W | Malimpia River | 722 | Nirchio et al. [2] |
| 20220825002AT1 | MECN 5013 | *Astroblepus theresiae* | OQ445485 | -0.06124S -78.78892W | Nambillo River | 1216 | This study |
| 1389 | MEPN-I 19082 | *Astroblepus theresiae* | PV874241 | 0.350277N -79.67972W | Cucaracha River | 529 | This study |
| 20200902AV1 | MECN 4954 | *Astroblepus sp.* | OQ420756 | -0.03298S -78.56861W | Pichán River | 2003 | This study |
| 20210908001AV1 | MECN 4953 | *Astroblepus sp.* | OQ420760 | 0.04078N -78.67732W | Alambi River | 1420 | This study |
| 20210908003AV4 | MECN 4957 | *Astroblepus sp.* | OQ420762 | -0.02298S -78.57311W | Pichán River | 1730 | This study |
| TAN | - | *Astroblepus sp.* | PV867025 | -0.01202S, -78.68958 | Tandayapa | 2200 | This study |

**References:**

1. Escobar Camacho D, Barragán KS, Guayasamin JM, Gavilanes G, Encalada AC. New records of native and introduced fish species in a river basin of Western Ecuador, the Chocó-Darien Ecoregion, using DNA barcoding. Aguirre WE, editor. PLoS One [Internet]. 2024 Mar 8;19(3):e0298970. Available from: https://dx.plos.org/10.1371/journal.pone.0298970

2. Nirchio M, Oliveira C, de Bello Cioffi M, Sassi FMC, Rizzi FP, Benavides SWN, et al. Integrative morphological, cytogenetic and molecular characterization of the Andean climbing catfish Astroblepus mindoensis (Regan, 1916) (Siluriformes: Astroblepidae). J Fish Biol [Internet]. 2024 Oct 9;(January):1–13. Available from: https://onlinelibrary.wiley.com/doi/10.1111/jfb.15924
