# Supplementary material for: Cryptic diversity in Astroblepus (Siluriformes: Astroblepidae): Integrative taxonomy reveals evolutionary complexity in the Esmeraldas River Basin, Ecuador
Source: PLoS One. 2026 Apr 22;21(4):e0343879. doi: 10.1371/journal.pone.0343879 (PMC13102232; doi:10.1371/journal.pone.0343879)
Supplement: S3 Table — (DOCX) [file pone.0343879.s005.docx]

**S5 Table.** GenBank sequences of *Astroblepus* used in this study.

| **Taxon** | **GenBank accession #** | **Country** | **References** |
| --- | --- | --- | --- |
| *Astroblepus* sp. A | HM049061.1 | Perú | Schaefer et al. [1] |
| *Astroblepus* sp. B | HM049065.1 | Perú | Schaefer et al. [1] |
| *Astroblepus* sp. B | HM049068.1 | Perú | Schaefer et al. [1] |
| *Astroblepus* sp. B | HM049064.1 | Perú | Schaefer et al. [1] |
| *Astroblepus* sp. B | HM049066.1 | Perú | Schaefer et al. [1] |
| *Astroblepus* sp. B | HM049054.1 | Perú | Schaefer et al. [1] |
| *Astroblepus* sp. B | HM049055.1 | Perú | Schaefer et al. [1] |
| *Astroblepus* sp. B | HM049059.1 | Perú | Schaefer et al. [1] |
| *Astroblepus* sp. C | HM049070.1 | Perú | Schaefer et al. [1] |
| *Astroblepus* sp. C | HM049067.1 | Perú | Schaefer et al. [1] |
| *Astroblepus* sp. C | HM049062.1 | Perú | Schaefer et al. [1] |
| *Astroblepus* sp. C | HM049069.1 | Perú | Schaefer et al. [1] |
| *Astroblepus* sp. C | HM049060.1 | Perú | Schaefer et al. [1] |
| *Astroblepus* sp. C | HM049071.1 | Perú | Schaefer et al. [1] |
| *Astroblepus* sp. C | HM049063.1 | Perú | Schaefer et al. [1] |
| *Astroblepus* sp. D | HM049050.1 | Perú | Schaefer et al. [1] |
| *Astroblepus* sp. D | HM049072.1 | Perú | Schaefer et al. [1] |
| *Astroblepus* sp. D | HM049049.1 | Perú | Schaefer et al. [1] |
| *Astroblepus* sp. E | HM049051.1 | Perú | Schaefer et al. [1] |
| *Astroblepus* sp. E | HM049057.1 | Perú | Schaefer et al. [1] |
| *Astroblepus* sp. E | HM049039.1 | Perú | Schaefer et al. [1] |
| *Astroblepus* sp. E | HM049047.1 | Perú | Schaefer et al. [1] |
| *Astroblepus* sp. F | HM049073.1 | Perú | Schaefer et al. [1] |
| *Astroblepus* sp. F | HM049053.1 | Perú | Schaefer et al. [1] |
| *Astroblepus* sp. F | HM049056.1 | Perú | Schaefer et al. [1] |
| *Astroblepus* sp. F | HM049046.1 | Perú | Schaefer et al. [1] |
| *Astroblepus* sp. F | HM049041.1 | Perú | Schaefer et al. [1] |
| *Astroblepus* sp. F | HM049045.1 | Perú | Schaefer et al. [1] |
| *Astroblepus* sp. H | HM049037.1 | Perú | Schaefer et al. [1] |
| *Astroblepus* sp. H | HM049038.1 | Perú | Schaefer et al. [1] |
| *Astroblepus* sp. H | HM049043.1 | Perú | Schaefer et al. [1] |
| *Astroblepus* sp. I | HM049044.1 | Perú | Schaefer et al. [1] |
| *Astroblepus* sp. 18 | MN542028.1 | Ecuador | Ochoa et al. [2] |
| *Astroblepus* sp. 18 | MN542029.1 | Ecuador | Ochoa et al. [2] |
| *Astroblepus* sp. 18 | MN542030.1 | Ecuador | Ochoa et al. [2] |
| *Astroblepus* sp. 19 | MN542025.1 | Ecuador | Ochoa et al. [2] |
| *Astroblepus* sp. 19 | MN542026.1 | Ecuador | Ochoa et al. [2] |
| *Astroblepus* sp. 19 | MN542027.1 | Ecuador | Ochoa et al. [2] |
| *Astroblepus caquetae* | MN542031.1 | Colombia | Ochoa et al. [2] |
| *Astroblepus caquetae* | MN542032.1 | Colombia | Ochoa et al. [2] |
| *Astroblepus caquetae* | MN542033.1 | Colombia | Ochoa et al. [2] |
| *Astroblepus caquetae* | MN542034.1 | Colombia | Ochoa et al. [2] |
| *Astroblepus caquetae* | MN542035.1 | Colombia | Ochoa et al. [2] |
| *Astroblepus grixalvii* group | MN542048.1 | Colombia | Ochoa et al. [2] |
| *Astroblepus grixalvii* group | MN542049.1 | Colombia | Ochoa et al. [2] |
| *Astroblepus grixalvii* group | MN542050.1 | Colombia | Ochoa et al. [2] |
| *Astroblepus grixalvii* group | MN542053.1 | Colombia | Ochoa et al. [2] |
| *Astroblepus grixalvii* group | MN542055.1 | Colombia | Ochoa et al. [2] |
| *Astroblepus grixalvii* group | MN542057.1 | Colombia | Ochoa et al. [2] |
| *Astroblepus grixalvii* group | MN542056.1 | Colombia | Ochoa et al. [2] |
| *Astroblepus grixalvii* group | MN542062.1 | Colombia | Ochoa et al. [2] |
| *Astroblepus grixalvii* group | MN542066.1 | Colombia | Ochoa et al. [2] |
| *Astroblepus grixalvii* group | MN542065.1 | Colombia | Ochoa et al. [2] |
| *Astroblepus grixalvii* group | MN542063.1 | Colombia | Ochoa et al. [2] |
| *Astroblepus grixalvii* group | MN542068.1 | Colombia | Ochoa et al. [2] |
| *Astroblepus grixalvii* group | MN542067.1 | Colombia | Ochoa et al. [2] |
| *Astroblepus grixalvii* group | MN542071.1 | Colombia | Ochoa et al. [2] |
| *Astroblepus grixalvii* group | MN542070.1 | Colombia | Ochoa et al. [2] |
| *Astroblepus grixalvii* group | MN542064.1 | Colombia | Ochoa et al. [2] |
| *Astroblepus grixalvii* group | MN542059.1 | Colombia | Ochoa et al. [2] |
| *Astroblepus grixalvii* group | MN542060.1 | Colombia | Ochoa et al. [2] |
| *Astroblepus grixalvii* group | MN542074.1 | Colombia | Ochoa et al. [2] |
| *Astroblepus grixalvii* group | MN542061.1 | Colombia | Ochoa et al. [2] |
| *Astroblepus grixalvii* group | MN542073.1 | Colombia | Ochoa et al. [2] |
| *Astroblepus grixalvii* group | MN542058.1 | Colombia | Ochoa et al. [2] |
| *Astroblepus grixalvii* group | MN542054.1 | Colombia | Ochoa et al. [2] |
| *Astroblepus grixalvii* group | MN542052.1 | Colombia | Ochoa et al. [2] |
| *Astroblepus grixalvii* group | MN542094.1 | Colombia | Ochoa et al. [2] |
| *Astroblepus grixalvii* group | MN542072.1 | Colombia | Ochoa et al. [2] |
| *Astroblepus grixalvii* group | MN542095.1 | Colombia | Ochoa et al. [2] |
| *Astroblepus grixalvii* group | MN542092.1 | Colombia | Ochoa et al. [2] |
| *Astroblepus grixalvii* group | MN542090.1 | Colombia | Ochoa et al. [2] |
| *Astroblepus grixalvii* group | MN542077.1 | Colombia | Ochoa et al. [2] |
| *Astroblepus grixalvii* group | MN542091.1 | Colombia | Ochoa et al. [2] |
| *Astroblepus grixalvii* group | MN542086.1 | Colombia | Ochoa et al. [2] |
| *Astroblepus grixalvii* group | MN542093.1 | Colombia | Ochoa et al. [2] |
| *Astroblepus grixalvii* group | MN542085.1 | Colombia | Ochoa et al. [2] |
| *Astroblepus grixalvii* group | MN542083.1 | Colombia | Ochoa et al. [2] |
| *Astroblepus grixalvii* group | MN542081.1 | Colombia | Ochoa et al. [2] |
| *Astroblepus grixalvii* group | MN542080.1 | Colombia | Ochoa et al. [2] |
| *Astroblepus grixalvii* group | MN542088.1 | Colombia | Ochoa et al. [2] |
| *Astroblepus grixalvii* group | MN542076.1 | Colombia | Ochoa et al. [2] |
| *Astroblepus grixalvii* group | MN542084.1 | Colombia | Ochoa et al. [2] |
| *Astroblepus grixalvii* group | MN542089.1 | Colombia | Ochoa et al. [2] |
| *Astroblepus grixalvii* group | MN542079.1 | Colombia | Ochoa et al. [2] |
| *Astroblepus grixalvii* group | MN542078.1 | Colombia | Ochoa et al. [2] |
| *Astroblepus grixalvii* group | MN542082.1 | Colombia | Ochoa et al. [2] |
| *Astroblepus grixalvii* group | MN542075.1 | Colombia | Ochoa et al. [2] |
| *Astroblepus grixalvii* group | MN542087.1 | Colombia | Ochoa et al. [2] |
| *Astroblepus mariae* | MN542173.1 | Colombia | Ochoa et al. [2] |
| *Astroblepus mariae* | MN542175.1 | Colombia | Ochoa et al. [2] |
| *Astroblepus mariae* | MN542174.1 | Colombia | Ochoa et al. [2] |
| *Astroblepus mariae* | MN542179.1 | Colombia | Ochoa et al. [2] |
| *Astroblepus mariae* | MN542178.1 | Colombia | Ochoa et al. [2] |
| *Astroblepus mariae* | MN542177.1 | Colombia | Ochoa et al. [2] |
| *Astroblepus mariae* | MN542176.1 | Colombia | Ochoa et al. [2] |
| *Astroblepus verai* | MN542039.1 | Colombia | Ochoa et al. [2] |
| *Astroblepus verai* | MN542038.1 | Colombia | Ochoa et al. [2] |
| *Astroblepus cacharas* | MN542042.1 | Colombia | Ochoa et al. [2] |
| *Astroblepus cacharas* | MN542043.1 | Colombia | Ochoa et al. [2] |
| *Astroblepus cacharas* | MN542041.1 | Colombia | Ochoa et al. [2] |
| *Astroblepus cacharas* | MN542040.1 | Colombia | Ochoa et al. [2] |
| *Astroblepus curitiensis* | MN542166.1 | Colombia | Ochoa et al. [2] |
| *Astroblepus curitiensis* | MN542168.1 | Colombia | Ochoa et al. [2] |
| *Astroblepus curitiensis* | MN542167.1 | Colombia | Ochoa et al. [2] |
| *Astroblepus curitiensis* | MN542162.1 | Colombia | Ochoa et al. [2] |
| *Astroblepus curitiensis* | MN542161.1 | Colombia | Ochoa et al. [2] |
| *Astroblepus curitiensis* | MN542164.1 | Colombia | Ochoa et al. [2] |
| *Astroblepus curitiensis* | MN542163.1 | Colombia | Ochoa et al. [2] |
| *Astroblepus curitiensis* | MN542165.1 | Colombia | Ochoa et al. [2] |
| *Astroblepus homodon* | MN542155.1 | Colombia | Ochoa et al. [2] |
| *Astroblepus homodon* | MN542157.1 | Colombia | Ochoa et al. [2] |
| *Astroblepus homodon* | MN542156.1 | Colombia | Ochoa et al. [2] |
| *Astroblepus homodon* | MN542160.1 | Colombia | Ochoa et al. [2] |
| *Astroblepus homodon* | MN542159.1 | Colombia | Ochoa et al. [2] |
| *Astroblepus homodon* | MN542144.1 | Colombia | Ochoa et al. [2] |
| *Astroblepus homodon* | MN542154.1 | Colombia | Ochoa et al. [2] |
| *Astroblepus homodon* | MN542158.1 | Colombia | Ochoa et al. [2] |
| *Astroblepus homodon* | MN542145.1 | Colombia | Ochoa et al. [2] |
| *Astroblepus homodon* | MN542146.1 | Colombia | Ochoa et al. [2] |
| *Astroblepus homodon* | MN542150.1 | Colombia | Ochoa et al. [2] |
| *Astroblepus homodon* | MN542149.1 | Colombia | Ochoa et al. [2] |
| *Astroblepus homodon* | MN542147.1 | Colombia | Ochoa et al. [2] |
| *Astroblepus homodon* | MN542151.1 | Colombia | Ochoa et al. [2] |
| *Astroblepus homodon* | MN542148.1 | Colombia | Ochoa et al. [2] |
| *Astroblepus homodon* | MN542153.1 | Colombia | Ochoa et al. [2] |
| *Astroblepus homodon* | MN542152.1 | Colombia | Ochoa et al. [2] |
| *Astroblepus nicefori* | MN542109.1 | Colombia | Ochoa et al. [2] |
| *Astroblepus nicefori* | MN542112.1 | Colombia | Ochoa et al. [2] |
| *Astroblepus nicefori* | MN542111.1 | Colombia | Ochoa et al. [2] |
| *Astroblepus* cf. *regani* | MN542108.1 | Ecuador | Ochoa et al. [2] |
| *Astroblepus* cf. *regani* | MN542107.1 | Ecuador | Ochoa et al. [2] |
| *Astroblepus* cf. *regani* | MN542105.1 | Ecuador | Ochoa et al. [2] |
| *Astroblepus* cf. *regani* | MN542106.1 | Ecuador | Ochoa et al. [2] |
| *Astroblepus itae* | MN542098.1 | Colombia | Ochoa et al. [2] |
| *Astroblepus ardiladuartei* | MN542100.1 | Colombia | Ochoa et al. [2] |
| *Astroblepus ardiladuartei* | MN542099.1 | Colombia | Ochoa et al. [2] |
| *Astroblepus onzagaensis* | MN542101.1 | Colombia | Ochoa et al. [2] |
| *Astroblepus onzagaensis* | MN542103.1 | Colombia | Ochoa et al. [2] |
| *Astroblepus onzagaensis* | MN542102.1 | Colombia | Ochoa et al. [2] |
| *Astroblepus* aff. *micrescens* | MN542118.1 | Colombia | Ochoa et al. [2] |
| *Astroblepus* aff. *micrescens* | MN542116.1 | Colombia | Ochoa et al. [2] |
| *Astroblepus* aff. *micrescens* | MN542117.1 | Colombia | Ochoa et al. [2] |
| *Astroblepus* aff. *trifasciatus* | MN542129.1 | Colombia | Ochoa et al. [2] |
| *Astroblepus* aff. *trifasciatus* | MN542136.1 | Colombia | Ochoa et al. [2] |
| *Astroblepus* aff. *trifasciatus* | MN542134.1 | Colombia | Ochoa et al. [2] |
| *Astroblepus* aff. *trifasciatus* | MN542130.1 | Colombia | Ochoa et al. [2] |
| *Astroblepus* aff. *trifasciatus* | MN542137.1 | Colombia | Ochoa et al. [2] |
| *Astroblepus* aff. *trifasciatus* | MN542126.1 | Colombia | Ochoa et al. [2] |
| *Astroblepus* aff. *trifasciatus* | MN542141.1 | Colombia | Ochoa et al. [2] |
| *Astroblepus* aff. *trifasciatus* | MN542131.1 | Colombia | Ochoa et al. [2] |
| *Astroblepus* aff. *trifasciatus* | MN542125.1 | Colombia | Ochoa et al. [2] |
| *Astroblepus* aff. *trifasciatus* | MN542140.1 | Colombia | Ochoa et al. [2] |
| *Astroblepus* aff. *trifasciatus* | MN542133.1 | Colombia | Ochoa et al. [2] |
| *Astroblepus* aff. *trifasciatus* | MN542128.1 | Colombia | Ochoa et al. [2] |
| *Astroblepus* aff. *trifasciatus* | MN542132.1 | Colombia | Ochoa et al. [2] |
| *Astroblepus* aff. *trifasciatus* | MN542127.1 | Colombia | Ochoa et al. [2] |
| *Astroblepus* aff. *trifasciatus* | MN542138.1 | Colombia | Ochoa et al. [2] |
| *Astroblepus* aff. *trifasciatus* | MN542139.1 | Colombia | Ochoa et al. [2] |
| *Astroblepus* aff. *trifasciatus* | MN542135.1 | Colombia | Ochoa et al. [2] |
| *Astroblepus verai* | PQ233888.1 | Colombia | Jiménez-Segura et al. [3] |
| *Astroblepus cacharas* | PQ233870.1 | Colombia | Jiménez-Segura et al. [3] |
| *Astroblepus itae* | PQ233869.1 | Colombia | Jiménez-Segura et al. [3] |
| *Astroblepus homodon* | PQ233832.1 | Colombia | Jiménez-Segura et al. [3] |
| *Astroblepus nicefori* | PQ233786.1 | Colombia | Jiménez-Segura et al. [3] |
| *Astroblepus nicefori* | PQ233785.1 | Colombia | Jiménez-Segura et al. [3] |
| *Astroblepus dux* | PQ233754.1 | Colombia | Jiménez-Segura et al. [3] |
| *Astroblepus dux* | PQ233304.1 | Colombia | Jiménez-Segura et al. [3] |
| *Astroblepus dux* | PQ233302.1 | Colombia | Jiménez-Segura et al. [3] |
| *Astroblepus dux* | PQ233303.1 | Colombia | Jiménez-Segura et al. [3] |
| *Astroblepus dux* | PQ233273.1 | Colombia | Jiménez-Segura et al. [3] |
| *Astroblepus dux* | PQ233209.1 | Colombia | Jiménez-Segura et al. [3] |
| *Astroblepus dux* | PQ233208.1 | Colombia | Jiménez-Segura et al. [3] |
| *Astroblepus dux* | PQ232927.1 | Colombia | Jiménez-Segura et al. [3] |
| *Astroblepus dux* | PQ232912.1 | Colombia | Jiménez-Segura et al. [3] |
| *Astroblepus dux* | PQ232883.1 | Colombia | Jiménez-Segura et al. [3] |
| *Astroblepus dux* | PQ232874.1 | Colombia | Jiménez-Segura et al. [3] |
| *Astroblepus dux* | PQ232849.1 | Colombia | Jiménez-Segura et al. [3] |
| *Astroblepus dux* | PQ232843.1 | Colombia | Jiménez-Segura et al. [3] |
| *Astroblepus dux* | PQ232830.1 | Colombia | Jiménez-Segura et al. [3] |
| *Astroblepus dux* | PQ232814.1 | Colombia | Jiménez-Segura et al. [3] |
| *Astroblepus dux* | PQ232813.1 | Colombia | Jiménez-Segura et al. [3] |
| *Astroblepus dux* | PQ232812.1 | Colombia | Jiménez-Segura et al. [3] |
| *Astroblepus dux* | PQ232808.1 | Colombia | Jiménez-Segura et al. [3] |
| *Astroblepus dux* | PQ232797.1 | Colombia | Jiménez-Segura et al. [3] |

**References:**

1. Schaefer SA, Chakrabarty P, Geneva AJ, Sabaj Pérez MH. Nucleotide sequence data confirm diagnosis and local endemism of variable morphospecies of Andean astroblepid catfishes (Siluriformes: Astroblepidae). Zool J Linn Soc [Internet]. 2011 May;162(1):90–102. Available from: https://academic.oup.com/zoolinnean/article-lookup/doi/10.1111/j.1096-3642.2010.00673.x

2. Ochoa LE, Melo BF, García-Melo JE, Maldonado-Ocampo JA, Souza CS, Albornoz-Garzón JG, et al. Species delimitation reveals an underestimated diversity of Andean catfishes of the family Astroblepidae (Teleostei: Siluriformes). Neotrop Ichthyol [Internet]. 2020;18(4):1–19. Available from: http://www.scielo.br/scielo.php?script=sci_arttext&pid=S1679-62252020000400205&tlng=en

3. Jiménez-Segura LF, Restrepo-Segura D, Ospina-Pabon J, Castellanos-Mejia M, Valencia-Rodriguez D, Galeano A, et al. GenBank. 2024 [cited 2024 Oct 17]. Fish databases for improving their conservation in Colombia. Available from: https://www.ncbi.nlm.nih.gov/bioproject/PRJNA1040268
