# Supplementary material for: Cryptic diversity in Astroblepus (Siluriformes: Astroblepidae): Integrative taxonomy reveals evolutionary complexity in the Esmeraldas River Basin, Ecuador
Source: PLoS One. 2026 Apr 22;21(4):e0343879. doi: 10.1371/journal.pone.0343879 (PMC13102232; doi:10.1371/journal.pone.0343879)
Supplement: S4 Table — (DOCX) [file pone.0343879.s006.docx]

**S6 Table.** GenBank accession numbers of the outgroup

| **Outgroup species** | **GenBank accession #** |
| --- | --- |
| *Loricaria simillima* | MK861710 |
| *Lamontichthys stibaros* | HM049035.1 |
| *Pterygoplichthys multiradiatus* | KR491517.1 |
